# Intestinal microbiota mediates Enterotoxigenic *Escherichia coli*-induced diarrhea in piglets

**DOI:** 10.1186/s12917-018-1704-9

**Published:** 2018-12-05

**Authors:** Peng Bin, Zhiyi Tang, Shaojuan Liu, Shuai Chen, Yaoyao Xia, Jiaqi Liu, Hucong Wu, Guoqiang Zhu

**Affiliations:** 1 Joint International Research Laboratory of Agriculture and Agri-Product Safety of Ministry of Education of China, Jiangsu Co-innovation Center for Important Animal Infectious Diseases and Zoonoses, College of Veterinary Medicine, Yangzhou University, Yangzhou, China; 2 College of Animal Science, South China Agricultural University, Guangzhou, 510642 China; 3 Laboratory of Animal Nutrition and Health and Key Laboratory of Agro-Ecology, Institute of Subtropical Agriculture, The Chinese Academy of Sciences, Changsha, Hunan 410125 People’s Republic of China

**Keywords:** Diarrhea, Enterotoxigenic *Escherichia coli*, Microbiota, Microbiota transplantation, Piglet

## Abstract

**Background:**

Enterotoxigenic *Escherichia coli* (ETEC) causes diarrhea in humans, cows, and pigs. The gut microbiota underlies pathology of several infectious diseases yet the role of the gut microbiota in the pathogenesis of ETEC-induced diarrhea is unknown.

**Results:**

By using an ETEC induced diarrheal model in piglet, we profiled the jejunal and fecal microbiota using metagenomics and 16S rRNA sequencing. A jejunal microbiota transplantation experiment was conducted to determine the role of the gut microbiota in ETEC-induced diarrhea. ETEC-induced diarrhea influenced the structure and function of gut microbiota. Diarrheal piglets had lower *Bacteroidetes*: *Firmicutes* ratio and microbiota diversity in the jejunum and feces, and lower percentage of *Prevotella* in the feces, but higher *Lactococcus* in the jejunum and higher *Escherichia-Shigella* in the feces. The transplantation of the jejunal microbiota from diarrheal piglets to uninfected piglets leaded to diarrhea after transplantation. Microbiota transplantation experiments also supported the notion that dysbiosis of gut microbiota is involved in the immune responses in ETEC-induced diarrhea.

**Conclusion:**

We conclude that ETEC infection influences the gut microbiota and the dysbiosis of gut microbiota after ETEC infection mediates the immune responses in ETEC infection.

**Electronic supplementary material:**

The online version of this article (10.1186/s12917-018-1704-9) contains supplementary material, which is available to authorized users.

## Background

The intestinal microbiota is considered as a new “functional organ” as it regulates plentiful physiological functions of host, such as digestion [1], metabolism [2, 3], immunity [4, 5] and so on. Changes in the composition of intestinal microbiota are associated with a series diseases and dysfunctions, including inflammatory bowel disease [6], obesity [7], colorectal cancer [8] and type 2 diabetes [9]. What’s more, changes in the composition of intestinal microbiota also increase the intestinal susceptibility to infection, as the indigenous intestinal microbiota-mediated innate and adaptive defense is disrupted [10]. In contrast, the pathogenic infection in intestine also affects the composition of intestinal microbiota. For example, *Salmonella enterica* infection, which affects the intestine of poultry and causes intestinal inflammation, increases the relative abundance of *Lactobacillaceae* in the cecum of chicken [11]. Scores of metabolites are produced by the intestinal microbiota, and certain metabolites play the crucial role in the mediation of host physiological functions. For instance, indolepropionic acid, which produced by *Clostridium sporogenes*, can reinforce the intestinal barrier by engaging the pregnane X receptor [12]. Thus, changes in the composition of intestinal microbiota may associate with the pathogenesis of several infectious diseases.

Diarrhea in piglets is a typical multifactorial disease in the swine production, and it is also the main cause of piglet death. However, the etiology and epidemiology of diarrhea in piglets is very complicated, and the Enterotoxigenic *Escherichia coli* (ETEC) is the most common food-borne epidemical pathogen which causes diarrhea [13]. Mechanism of ETEC-induced diarrhea depends on the fimbrial adhesins and enterotoxin, which promotes the pathogen bacteria to adhere on the intestinal epithelial cells of piglets and lead to the fluid-electrolytes disturbance and acid-base imbalance of piglets, respectively [14, 15]. However, not all individuals infected with ETEC suffer from diarrhea [16]. Also, our previous investigation found that about 50% of piglets develop diarrhea after ETEC infection [17]. Whether the intestinal microbiota is related to the susceptibility of individuals and pigs to ETEC infection and development of diarrhea is unknown.

Previous investigations have shown that diarrhea is linked with changes of the intestinal microbiota composition [18–20]. However, osmotic diarrhea in humans also induces changes in microbial community structure [21], suggesting the alteration of gut microbiota may be the result of diarrhea. Thus, the cause and effect relationship between the changed intestinal microbiota and infectious diarrhea is unclear. This study was conducted to validate the hypothesis that the intestinal microbiota is changed in ETEC-induced diarrhea, and the intestinal microbiota is involved in the pathogenesis of ETEC-induced diarrhea.

## Methods

### Bacterial strains

The strain *Escherichia coli* W25K (O149:K91, K88 ac; LT, STb, EAST) was used in the current study, which was originally isolated from a diarrheal piglet [22].

### ETEC infection

This study was conducted according to the guidelines of College of Animal Science, South China Agricultural University. This study was approved by South China Agricultural University. The Establishment of animal diarrheal model was consistent with our previous study [17, 23]. In brief, fifty-one piglets (Landrace × Yorkshire; 18-days old) were obtained from our partner farm (ZhengDa Co., Chongqing, China). Meanwhile, 41 piglets were randomly selected to receive an oral inoculation with the ETEC W25K (10^10^ CFUs/ml) and the rest of 10 piglets received the orally inoculation with the same volume LB medium as control. All the piglets experienced a five-day inoculate administration, and the fecal consistency was observed daily. Piglets which challenged with LB medium were defined as control group; piglets which developed watery diarrhea were marked during the whole experiment, and the marked piglets were regarded as recovered piglets if they recovered from diarrhea, otherwise were considered as diarrheal piglets; piglets those challenged with ETEC but not suffered from diarrhea were regarded as resistant piglets. Fresh feces were collected from day 1 to day 5 (post infection) for all kinds of piglets. For diarrheal piglets, their fresh feces were collected before diarrhea after ETEC infection to consider as pre-diarrheal samples. Six control piglets (*n* = 6), six diarrheal piglets (*n* = 6), six recovered piglets (*n* = 6) and six resistant piglets (*n* = 6) were randomly selected and sacrificed by kalium chloratum injection at day 6. Jejunum contents were collected after ice-cold phosphate buffered saline (PBS; pH = 7.2–7.4) washing. The whole part (including epithelium, mucosa, submucosa, muscular and serosa) of jejunum samples (middle part, about 3 cm) also collected after the ice-cold PBS washing. Luminal contents, feces and jejunum were collected and stored at − 80 °C until processing.

### Microbiota transplantation experiment

Jejunal luminal contents from diarrheal piglets and uninfected piglets were collected and used for transplantation. The jejunal luminal contents were collected and mixed with phosphate buffered saline (PBS; pH = 7.2–7.4), and the final volume was adjusted to 50 ml per piglet. The mixed solution was vortexed at maximum speed for 3 min, and then centrifuged for 5 min at 500*g. The donor jejunal solution was orally infused to piglets with oro-gastric tube within 1 h after collection. The jejunal solution from diarrheal piglets was infused to uninfected piglets (*n* = 12). As controls, the jejunal solution from uninfected piglets was orally infused to uninfected piglets (*n* = 6). Piglets were orally transplanted for five consecutive days (day 1- day 6) with 20 ml jejunal solution per day. Piglets in control group were defined as control piglets; piglets developed watery diarrhea after transplantation were scarified and defined as transplanted diarrheal piglets; at day 6, piglets without diarrhea after transplantation were scarified and defined as transplanted non-diarrheal piglets. The fresh feces were collected from day 1 to day 5 after transplantation. At day 6, piglets were sacrificed by kalium chloratum injection for sample collection after electrical stunning. The jejunum contents were collected by ice-cold PBS washing and jejunum samples were collected after the ice-cold PBS washing. All the samples were store at − 80 °C for further analysis.

### 16S rRNA sequencing

The frozen jejunum contents and feces were thawed at the room temperate, and bacterial DNA was extracted by a commercial DNA stool kit (Qiagen, Hilden, Germany) according the manufacturer’s protocols. We measured the DNA concentration and purity with a NanoDrop ND-1000 instrument (NanoDrop Technologies Inc., Wilmington, DE, USA). Equal amounts of DNA from four different piglets were pooled to generate one common sample for each type of treatment. The following 16S rRNA gene amplification and pyrosequencing analysis were entrusted to a commercial company (Biotree, Shanghai, China), and the methodology and procedure were accordance with our previous study [24, 25].

### Metagenomics analysis

The DNA extraction was described as above, and equal amounts of DNA from three different piglets were pooled to generate one common sample for each type of treatment (Diarrhea, Recovery, Resistant and Control). The metagenomics analysis of jejunal content was consigned to the commercial company (BGI Life Tech Co., Ltd., Beijing, China). DNA library construction, sequencing, de novo assembly, taxonomic assignment, and gene functional classification were based on their previous work [26, 27]. The total data volume of high-quality reads for our each group was nearly 14 Gbp.

### RT-PCR

The mRNA expression of *Tlr*5, *Tlr*4 and *Lyz-*2 was performed by real-time quantitative PCR. Briefly, 100 mg frozen jejunal samples were pulverized in the liquid nitrogen and mixed into 1 ml Trizol (Invitrogen, USA), and the total RNA was extracted following the manufacturer’s protocols. The quality and concentration were detected by a NanoDrop ND-1000 instrument (NanoDrop Technologies Inc., Wilmington, DE, USA). Afterwards, we used the DNase I (Invitrogen, USA) and Superscript II reverse transcriptase (Invitrogen, USA) to produce complementary DNA. To normalise the expression levels of the target genes, β-actin was used as the internal control, and primers used in current study were referred to the previous studies. The RT-PCR was performed as our description in the ref. [28–30].

### Fecal bacteria analysis using real-time PCR

The protocol and the primers used for feces *Bacteroidetes* and *Firmicutes* abundance analysis was conducted as described previously [28, 29].

### Statistical analyses

Data in the current study are analyzed by the software Prime 6 and SPSS 22.0, and all the data are presented as means ± standard error of the mean (SEM). The methods of statistical analyses were performed as the previous study [24]. Significant differences were declared when *P* < 0.05.

## Results

### ETEC-induced diarrhea was associated with alterations in intestinal microbiota

**Fig. 1:**
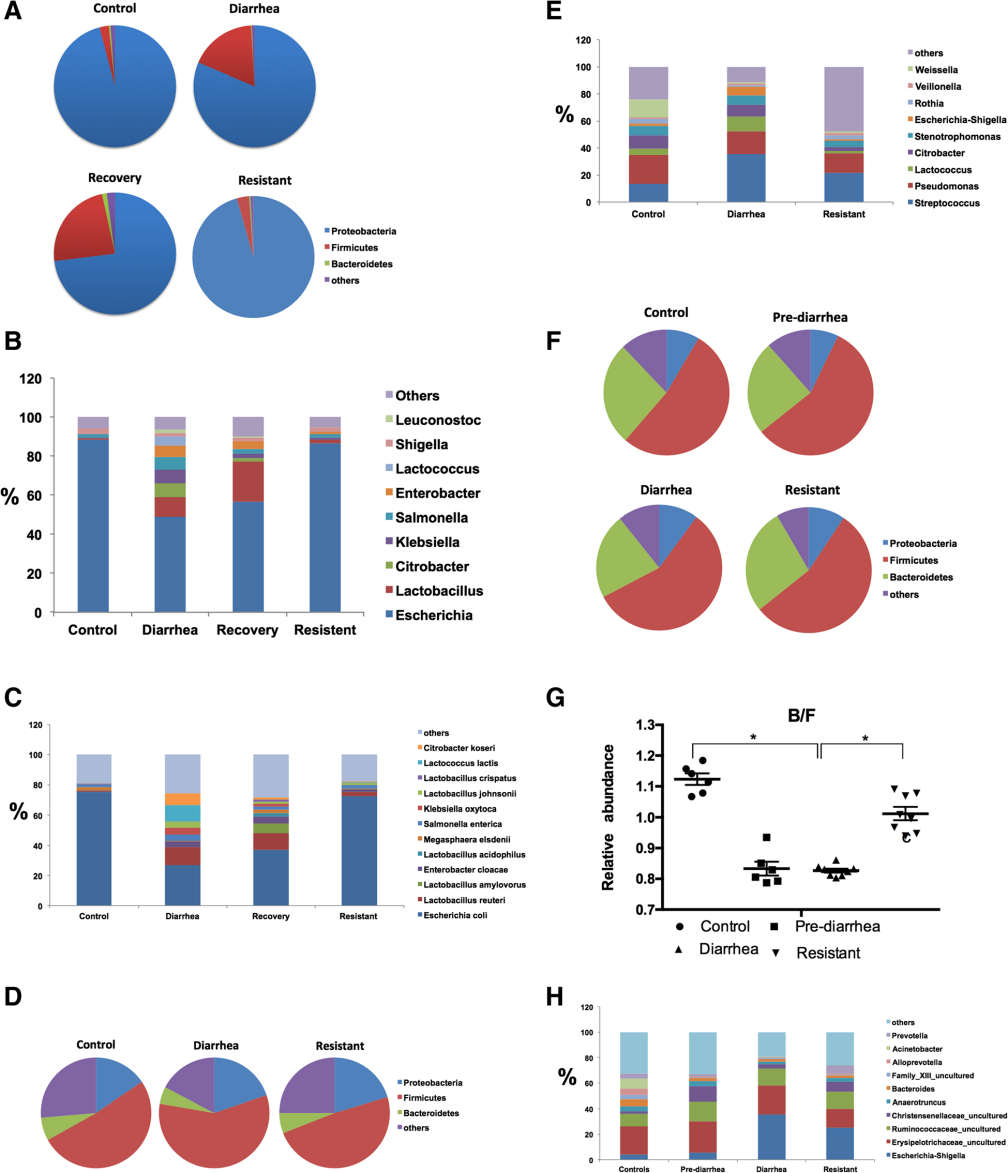
Gut microbiota in ETEC induced diarrhea. **a** The jejunal microbiota at the phylum level among controls, diarrheal piglets, recovered piglets and resistant piglets were analyzed using metagenomics. **b** The jejunal microbiota at the genus level were analyzed using metagenomics. **c** The jejunal microbiota at the species level were analyzed using metagenomics. **d** The jejunal microbiota at the phylum level among controls, diarrheal piglets and resistant piglets were analyzed using 16S rRNA sequencing. **e** The jejunal microbiota at the genus level were analyzed using 16S rRNA sequencing. **f** The fecal microbiota at the phylum level among controls, pre-diarrheal piglets, and resistant piglets were analyzed using 16S rRNA sequencing. **g** Real time-PCR analysis of the relative abundance of *Bacteroidetes* and *Firmicutes* in the feces among controls, pre-diarrheal piglets, and resistant piglets (*n* = 6; *: *p* < 0.05, one-way ANOVA). **h** The fecal microbiota at the genus level were analyzed using 16S rRNA sequencing. *N* = 3 before pooling for **a**, **b**, **c**; and *n* = 4 before pooling for **d**, **e**, **f**, **h**

We characterized the jejnunal microbiota in piglets using metagenomics sequencing (Fig. 1a). The two most abundant phyla in diarrheal piglets, accounting for approximately 99% of all assigned sequence reads were *Proteobacteria* (81%) and *Firmicutes* (18%). In piglets that recovered from diarrhea, they were *Proteobacteria* (73%) and *Firmicutes* (24%). For controls or resistant piglets, they were *Proteobacteria* (96%) and *Firmicutes* (2%), and *Proteobacteria* (96%) and *Firmicutes* (3%), respectively. At the genus level (Fig. 1b), the percentage of *Escherichia* (49% vs. 88%) was reduced in diarrheal piglets, while the relative abundance of *Lactobacillus* (10% vs. 0.6%), *Citrobacter* (7.1% vs. 0.3%), *Klebsiella* (6.8% vs. 0.7%), *Salmonella* (6.2% vs. 1.3%), *Enterobacter* (6.2% vs. 0.3%), *Lactococcus* (4.9% vs. 0.008%), and *Leuconostoc* (1.6% vs. 0.007%) was increased in diarrheal piglets compared to the controls (Fig. 1b). Compared to the resistant piglets, the percentage of *Escherichia* (49% vs. 86%) was reduced, while the relative abundance of *Lactobacillus* (10% vs. 1.8%), *Citrobacter* (7.1% vs. 0.3%), *Klebsiella* (6.8% vs. 0.8%), *Salmonella* (6.2% vs. 1.8%), *Enterobacter* (6.2% vs. 1.0%), *Lactococcus* (4.9% vs. 0.1%), and *Leuconostoc* (1.6% vs. 0.06%) was increased in diarrheal piglets (Fig. 1b). For recovered piglets, diarrheal piglets had higher percentage of *Escherichia* (57% vs. 49%) and *Lactobacillus* (20% vs. 10%), whereas they had the lower relative abundance of *Citrobacter* (1.3% vs. 7.1%), *Klebsiella* (2.8% vs. 6.8%), *Salmonella* (1.9% vs. 6.2%), *Enterobacter* (4.4% vs. 6.2%), *Lactococcus* (0.03% vs. 4.9%), and *Leuconostoc* (0.1% vs. 1.6%) (Fig. 1b). At the species level, compared to controls, diarrheal piglets had lower relative abundance of *Escherichia coli* (27% vs. 76%) and *Megasphaera elsdeii* (0.04% vs. 2.1%), and higher percentage of *Lactobacillus reuteri* (12% vs. 0.2%), *Enterobacter cloacae* (3.7% vs. 0.3%), *Klebsiella oxytoca* (4.2% vs. 0.01%), *Lactobacillus johnsonil* (4.2% vs. 0.1%), *Lactococcus lactis* (11% vs. 0.001%) and *Citrobacter koseri* (7.8% vs. 0.3%) (Fig.1c). Compared to resistant piglets, diarrheal piglets had lower percentage of *Escherichia coli* (27% vs. 72%), but higher percentage of *Lactobacillus reuteri* (12% vs. 2.6%), *Klebsiella oxytoca* (4.2% vs. 0.3%), *Lactococcus lactis* (11% vs. 0.4%) and *Citrobacter koseri* (7.8% vs. 0.4%) (Fig.1c). In recovered piglets, the percentage of *Escherichia coli* (37% vs. 27%) and *Megasphaera elsdeii* (2% vs. 0.04%) increased, while the percentage of *Salmonella enterica* (1.9% vs. 4.6%), *Klebsiella oxytoca* (1.8% vs. 4.2%), *Lactobacillus johnsonil* (1.6% vs. 4.3%), *Lactococcus lactis* (0.05% vs. 11%) and *Citrobacter koseri* (1.2% vs. 7.8%) decreased compared to diarrheal piglets (Fig.1c). Also, compared to diarrheal piglets, recovered piglets had higher relative abundance of *Lactobacillus amylovorus* (6.3% vs. 0.2%), *Lactobacillus acidophilus* (2.6% vs. 0.09%) and *Lactobacillus crispatus* (1.3% vs. 0.2%) (Fig.1c).

Previous studies have shown that diarrhea may result in a lower *Bacteroidetes*:*Firmicutes* ratio because the diarrhea may create a more suitable environment for the survival and growth of *Firmicutes* as compared with *Bacteroidetes* [18, 31–33]. The *Bacteroidetes*:*Firmicutes* ratio was 0.01 in diarrheal piglets, compared with 0.18 in control and 0.10 in resistant piglets. And the *Bacteroidetes*:*Firmicutes* ratio was 0.05 in recovered piglets (Table 1).

**Table 1:** Bacteroidetes:Firmicutes ratios

Piglet	Control	Resistant	Diarrhea	Recovery	Pre-diarrhea
1st - Jejunum	0.18	0.10	0.01	0.05	
2nd - Jejunum	0.13	0.12	0.08		
2nd - Feces	0.50	0.50	0.38		0.42

The intestinal microbiota in the jejunum among controls, resistant piglets, diarrheal piglets and recovered piglets were analyzed using metagenomics (1st, *n* = 3 before pooling) or using16S rRNA sequencing (2nd, *n* = 4 before pooling). The microbial diversity in these feces among controls, resistant piglets, diarrheal piglets and pre-diarrheal piglets were analyzed using 16S rRNA sequencing (2nd, *n* = 4 before pooling)

The ratios of *Bacteroidetes*: *Firmicutes* were calculated based on the relative percentage of *Bacteroidetes* to the relative percentage of *Firmicutes*

The microbiota in jejunal luminal content and feces were also analyzed using 16S rRNA sequencing (Table 2). For microbiota in jejunal luminal content, both Shannon and Simpson indices demonstrated that the microbiota diversity in the jejunum of diarrheal piglets was lower than controls or resistant piglets (Table 2). Noticeably, the community richness of microbiota in the jejunum was similar among diarrheal piglets, resistant piglets and controls (Table 2). At the phylum level (Fig. 1d), the three most abundant phyla in jejunal luminal contents were *Firmicutes* (58%), *Proteobacteria* (20%) and *Bacteroidetes* (5%) in diarrheal piglets. For piglets in control or resistant groups, they were *Firmicutes* (51%), *Proteobacteria* (16%) and *Bacteroidetes* (7%), and *Firmicutes* (49%), *Proteobacteria* (20%) and *Bacteroidetes* (6%), respectively. At the genus level (Fig. 1e), the percentage of *Streptococcus* (35% vs. 13%), *Lactococcus* (10.5% vs. 4.9%) and *Escherichia-Shigella* (6.1% vs. 1.9%) were increased, while *Weissella* (1.1% vs. 13.3%) was decreased in diarrheal piglets, compared to controls. Compared to resistant piglets, the percentage of *Streptococcus* (35% vs. 21%), *Lactococcus* (10.5% vs. 2.0%) and *Escherichia-Shigella* (6.1% vs. 1.4%) were increased in diarrheal piglets (Fig. 1e). The *Bacteroidetes*:*Firmicutes* ratio was 0.08 in diarrheal piglets, compared with 0.13 in control and 0.12 in resistant piglets (Table 1).

**Table 2:** Comparison of phylotype coverage and diversity estimation of the 16S rRNA gene libraries at 97% similarity from the MiSeq analysis

Group	No. of reads	No. of OTU^a^	Coverage^b^	Richness estimator		Diversity index	
				Ace(95% CI)	Chao(95% CI)	Shannon (95% CI)	Simpson(95% CI)
Jejunum							
Control	22,244	148	99.85%	178 (162–210)	198 (168–271)	2.91 (2.89–2.93)	0.09 (0.095–0.099)
Diarrhea	8672	121	99.51%	199 (171–242)	183 (149–258)	2.67(2.64–2.7)	0.10(0.10–0.11)
Resistant	11,338	152	99.86%	161 (155–176)	161 (155–182)	3.35 (3.33–3.38)	0.069 (0.067–0.071)
Transplanted-ND	20,681	113	99.83%	186 (158–231)	166 (135–236)	2.64 (2.63–2.66)	0.117(0.115–0.119)
Transplanted-D	15,902	108	99.81%	137 (122–168)	130 (117–161)	2.21(2.18–2.23)	0.225 (0.219–0.231)
Feces							
Control	19,789	305	99.81%	326 (316–346)	326 (314–352)	3.85(3.83–3.88)	0.065 (0.063–0.067)
Pre-diarrhea	11,543	277	99.61%	308 (294–332)	305 (290–336)	3.71 (3.67–3.74)	0.076 (0.073–0.079)
Diarrhea	22,342	327	99.72%	372 (254–402)	392 (290–336)	2.94(2.91–2.97)	0.176 (0.172–0.18)
Resistant	17,855	288	99.75%	314 (302–335)	322 (304–360)	3.49(3.46–3.52)	0.094 (0.091–0.097)
Transplanted-ND	12,222	276	99.60%	309 (295–339)	325 (300–376)	3.9(3.87–3.93)	0.048(0.046–0.05)
Transplanted-D	11,545	295	99.43%	347 (327–381)	360 (330–416)	3.66(3.63–3.7)	0.075(0.072–0.078)

^a^The operational taxonomic units (OTUs) were defined at the 97% similarity level (*n* = 4 before pooling)

^b^The coverage percentage, richness estimators (ACE and Chao) and diversity indices (Shannon and Simpson) were calculated using the mothur program

Piglets after the development of watery diarrhea by transplantation were scarified and defined as transplanted diarrheal piglets (Transplanted-D). At day 6, piglets without diarrhea after transplantation were scarified and defined as transplanted non-diarrheal piglets (Transplanted-ND)

In feces, the microbiota diversity of diarrheal piglets was lower than pre-diarrheal piglets, resistant piglets and controls, while little difference about the community richness of microbiota was observed among these groups (Table 2). The three most abundant phyla were *Firmicutes*, *Bacteroidetes*, and *Proteobacteria* in all groups (Fig. 1f). *Bacteroidetes*:*Firmicutes* ratio in the feces was 0.38 for diarrheal piglets, while it was 0.42 for pre-diarrheal piglets, and 0.50 for control and resistant piglets (Table 1). Real-time PCR data also shown the ratio of *Bacteroidetes*:*Firmicutes* was lower (*P* < 0.05) in diarrheal piglets, compared to the controls and resistant piglets (*n* = 4) (Fig. 1g). At the genus level (Fig. 1h), from controls, pre-diarrheal piglets to diarrheal piglets, the percentage of *Escherichia-Shigella* (3.8, 5.5 to 35.3%) increased, while *Prevotella* (4.2, 1.7 to 0.2%) decreased. Compared to resistant piglets, diarrheal piglets had higher relative abundance of *Escherichia-Shigella* (35.3% vs. 24.9%), while lower percentage of *Prevotella* (0.2% vs. 6.7%) (Fig. 1h).

### Jejunal microbiota mediates diarrhea

**Fig. 2:**
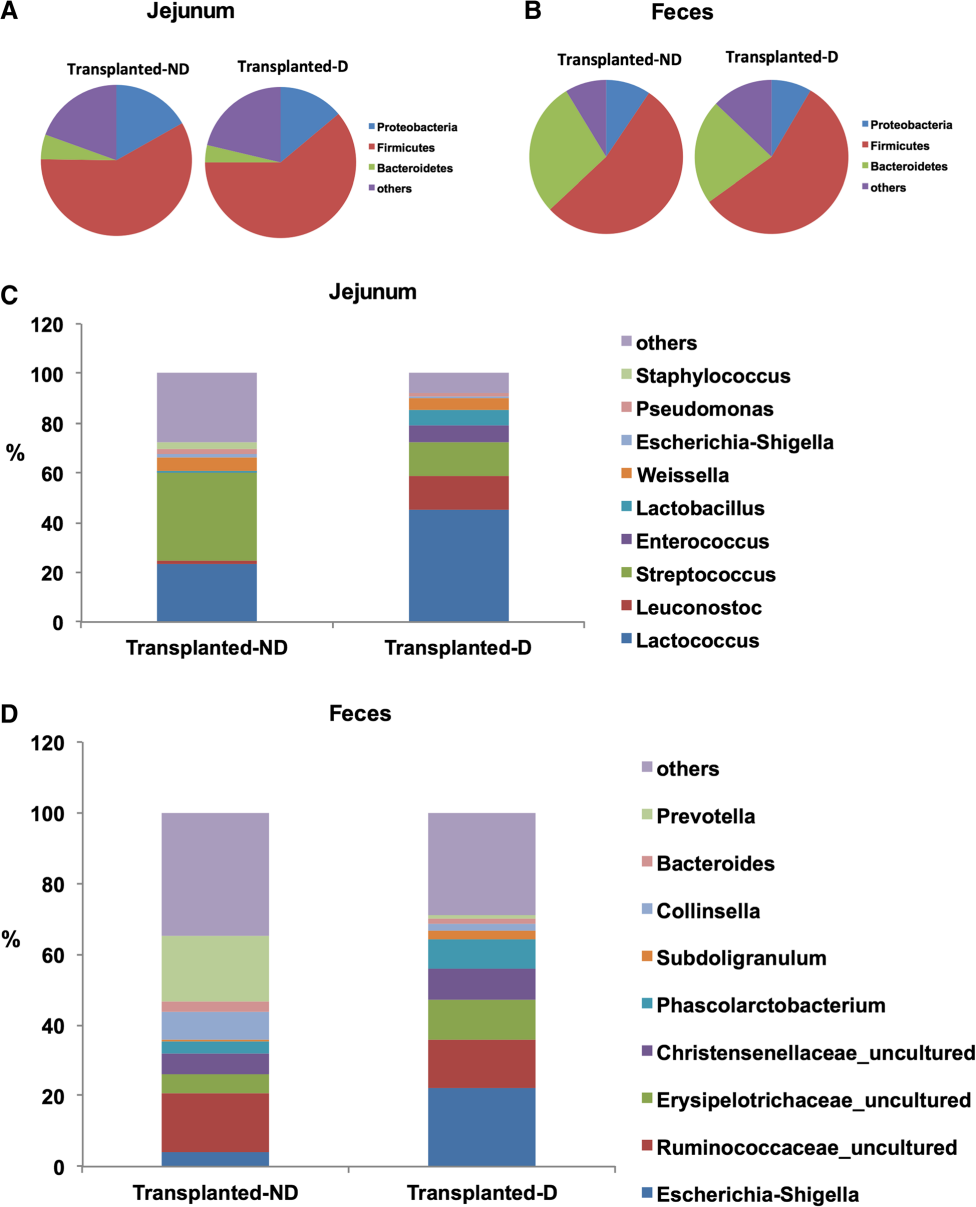
Gut microbiota in transplanted diarrhea. **a** The jejunal microbiota at the phylum level were analyzed using 16S rRNA sequencing. **b** Fecal microbiota at the phylum level were analyzed using 16S rRNA sequencing. **c** The jejunal microbiota at the genus level were analyzed using 16S rRNA sequencing. **d** Fecal microbiota at the genus level were analyzed using 16S rRNA sequencing. Transplanted-D: recipient piglets that experienced diarrhea after microbiota transplantation from diarrheal piglets. Transplanted-ND: recipient piglets that did not experience diarrhea after microbiota transplantation from diarrheal piglets. *N* = 4 before pooling for **a**, **b**, **c**, and **d**

To explore the cause and effect relationship between change in the gut microbiota and diarrhea, we conducted a jejunal microbiota transplantation experiment. As controls, we transplanted the jejunal microbiota from uninfected piglets (*n* = 4) to uninfected piglets,and we found that no transplanted piglets experienced diarrhea. We then transplanted the jejunal microbiota from diarrheal piglets to uninfected piglets, and 50% of these piglets exhibited diarrhea (*n* = 5). Compared to the non-diarrheal piglets after transplantation, diarrheal piglets had lower microbiota diversity in the jejunum and feces (Table 2). The relative abundance of *Bacteroidetes* decreased in diarrheal piglets, but the relative abundance of *Firmicutes* increased in the jejunum and feces (Fig.2 a-b, Table 3). For microbiota in the jejunum, diarrheal piglets had higher percentage of *Lactococcus* (45% vs. 23%), *Leuconostoc* (14% vs. 1.7%), *Enterococcus* (7% vs. 0.5%) and *Lactobacillus* (6% vs. 0.7%), but lower *Streptococcus* (13% vs. 34%) than the non-diarrheal piglets (Fig.2c). In the feces, diarrheal piglets had higher percentage of *Escherichia-Shigella* (22% vs. 4%) and *Erysipelotrichaceae-*uncultured (11% vs. 5%), but lower *Prevotella* (1% vs. 18%) than the non-diarrheal piglets (Fig.2d).

**Table 3:** *Bacteroidetes:Firmicutes* ratios from the MiSeq analysis

Piglets	Jejunum	Feces
Transplantation-D	0.06	0.39
Transplantation- ND	0.09	0.53

The microbial diversity in the piglet jejunum and feces were analyzed using 16S rRNA sequencing (*n* = 4 before pooling)*.* The ratios of *Bacteroidetes*: *Firmicutes* were calculated based on the relative percentage of *Bacteroidetes* to the relative percentage of *Firmicutes*

Piglets after the development of watery diarrhea by transplantation were defined as transplanted diarrheal piglets (Transplanted-D). At day 6, piglets without diarrhea after transplantation were defined as transplanted non-diarrheal piglets (Transplanted-ND)

### The protein repertoire and pathways impacted by ETEC induced diarrhea

Metagenomic sequences were annotated against the Kyoto Encyclopedia of Genes and Genomes (KEGG) databases. At KEGG level one, a total of 6 KEGG entries were identified (Additional file 1: S1), including metabolism, environmental information processing, genetic information processing, cellular processes, human diseases and organismal systems. Diarrheal piglets shows decrease in cellular processes, compared to the control piglets (Table 4). At KEGG level two, a total of 37 KEGG entries were identified (Additional file 1: S2). The six most abundant changed KEGG were cell motility, biosynthesis of other secondary metabolites, excretory system, immune system diseases, immune system and circulatory system (Table 4). ETEC induced diarrhea reduced the cell motility and biosynthesis of other secondary metabolites in jejunal microbiota (Table 4). At KEGG level three, a total of 236 KEGG Orthology (KO) pathways were identified (Additional file 1: S3). Among the 83 most abundant changed KO, ETEC induced diarrhea decreased bacterial invasion of epithelial cells, bacterial motility proteins and flagellar assembly for jejunum microbiota (Table 4). Collectively, these results suggest that ETEC induced diarrhea may influence the function of jejunum microbiota.

**Table 4:** Different KEGG entries between Diarrheal piglets and Control piglets

Levels	Fold change (Diarrhea/Control)	Annotation
KEGG level 1		
	0.77	Cellular Processes
KEGG level 2		
	0.71	Cell Motility
	0.77	Biosynthesis of Other Secondary Metabolites
	1.28	Excretory System
	1.29	Immune System Diseases
	1.58	Immune System
	2.89	Circulatory System
KEGG level 3		
	0.31	N-Glycan biosynthesis
	0.34	Flavone and flavonol biosynthesis
	0.35	Bacterial invasion of epithelial cells
	0.40	Penicillin and cephalosporin biosynthesis
	0.42	beta-Lactam resistance
	0.43	Stilbenoid, diarylheptanoid and gingerol biosynthesis
	0.59	Bisphenol degradation
	0.62	Apoptosis
	0.62	Non-homologous end-joining
	0.63	Secretion system
	0.65	Adipocytokine signaling pathway
	0.67	Bacterial motility proteins
	0.68	Other glycan degradation
	0.69	Phenylalanine metabolism
	0.70	Isoquinoline alkaloid biosynthesis
	0.71	Biosynthesis of vancomycin group antibiotics
	0.71	Carbohydrate metabolism
	0.72	Lysosome
	0.73	Tropane, piperidine and pyridine alkaloid biosynthesis
	0.73	beta-Alanine metabolism
	0.73	Glycosaminoglycan degradation
	0.73	Replication, recombination and repair proteins
	0.75	Ethylbenzene degradation
	0.75	Nucleotide metabolism
	0.75	Geraniol degradation
	0.76	Polyketide sugar unit biosynthesis
	0.76	Bladder cancer
	0.77	Ribosome biogenesis in eukaryotes
	0.78	Vibrio cholerae pathogenic cycle
	0.78	Limonene and pinene degradation
	0.78	Dioxin degradation
	0.79	Glyoxylate and dicarboxylate metabolism
	0.79	Flagellar assembly
	0.79	Caprolactam degradation
	0.79	Energy metabolism
	1.21	Aminoacyl-tRNA biosynthesis
	1.21	Zeatin biosynthesis
	1.22	DNA replication
	1.22	Terpenoid backbone biosynthesis
	1.22	Homologous recombination
	1.24	Steroid biosynthesis
	1.24	Carotenoid biosynthesis
	1.24	D-Glutamine and D-glutamate metabolism
	1.24	Peptidoglycan biosynthesis
	1.24	Ribosome
	1.24	Ribosome
	1.24	Mismatch repair
	1.25	Nucleotide excision repair
	1.25	Pyrimidine metabolism
	1.26	Phenylpropanoid biosynthesis
	1.26	Proximal tubule bicarbonate reclamation
	1.26	Phosphotransferase system (PTS)
	1.27	Alzheimer’s disease
	1.28	Primary immunodeficiency
	1.28	MAPK signaling pathway - yeast
	1.29	Lysine biosynthesis
	1.31	Cyanoamino acid metabolism
	1.32	Fatty acid biosynthesis
	1.32	Ubiquitin system
	1.33	Pentose phosphate pathway
	1.34	Photosynthesis proteins
	1.36	Sphingolipid metabolism
	1.36	RNA transport
	1.36	Photosynthesis
	1.40	D-Alanine metabolism
	1.41	Butirosin and neomycin biosynthesis
	1.47	Histidine metabolism
	1.51	Restriction enzyme
	1.59	Glycerolipid metabolism
	1.90	Inositol phosphate metabolism
	2.00	Phosphonate and phosphinate metabolism
	2.01	Glycosphingolipid biosynthesis - globo series
	2.14	Sporulation
	2.23	Steroid hormone biosynthesis
	2.47	Linoleic acid metabolism
	2.86	Cardiac muscle contraction
	2.86	Parkinson’s disease
	4.95	Ether lipid metabolism
	6.59	Glycosphingolipid biosynthesis - ganglio series
	20.58	*Staphylococcus aureus* infection
	21.64	Primary bile acid biosynthesis
	21.64	Secondary bile acid biosynthesis
	70.47	Atrazine degradation

KEGG entries in the intestinal microbiota of the jejunum among diarrheal piglets, recovered piglets, controls, and resistant piglets were analyzed using metagenomics (*n* = 3 before pooling). The fold changes of KEGG entries at each levels were calculated based on the relative percentage of KEGG entries in diarrheal piglets to the relative percentage of KEGG entries in control piglets. Those KEGG entries with the values of fold change < 0.8 or > 1.2 are listed

### Jejunal microbiota mediates the immune inhibition on the jejunum

**Fig. 3:**
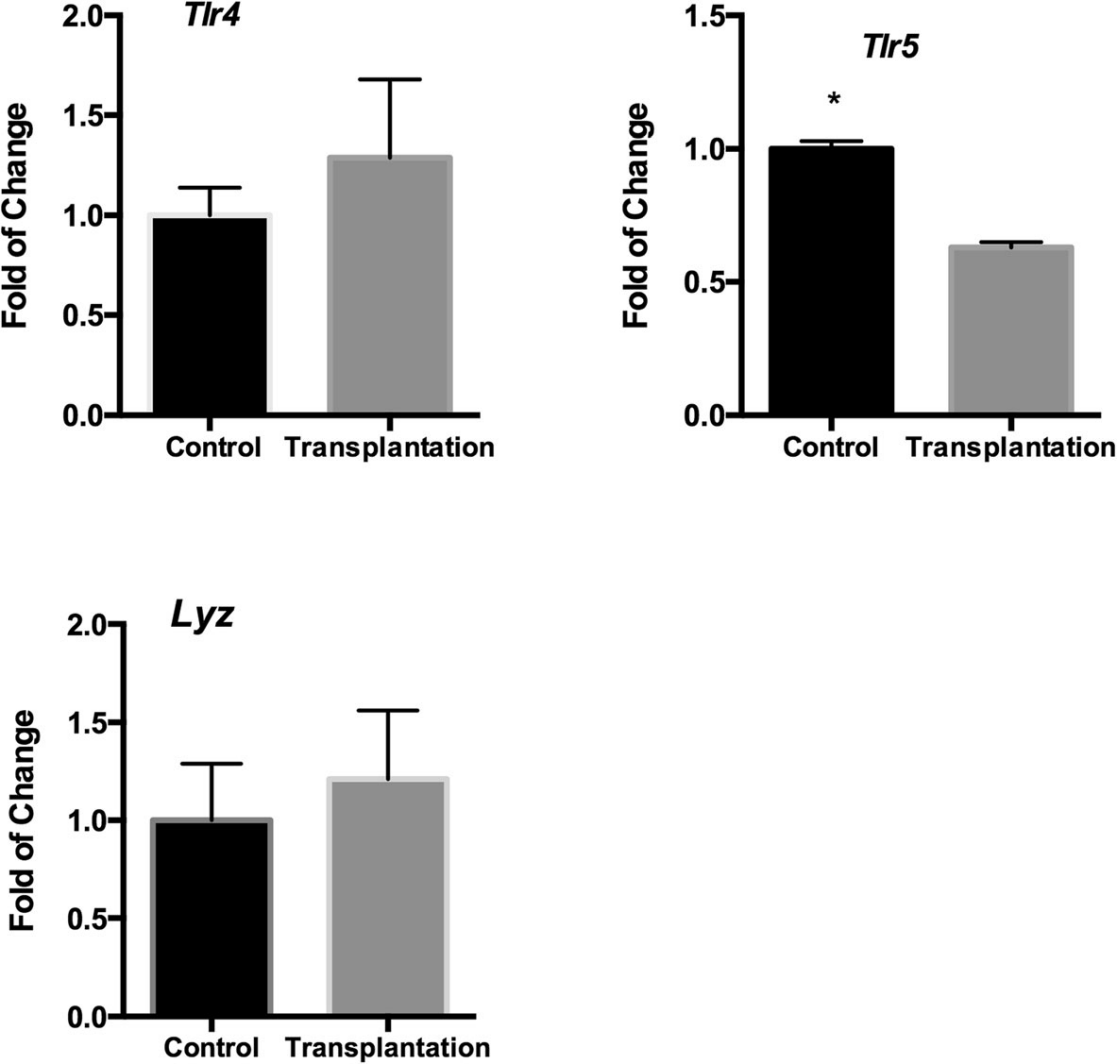
mRNA expression of innate immune genes after jejunal microbiota transplantation from normal (control) and diarrheal piglets (transplantation). (*n* = 4; *: *p* < 0.05, unpaired t test)

As shown in Table 4, diarrheal piglets suffered the dysbiosis of jejunal microbiota, including decreased cell motility and flagellar assembly (Table 4). This indicates that the inhibition of intestinal immunity in diarrheal piglets may come from the changed intestinal microbiota. To validate this, we transplanted the jejunal microbiota from diarrheal piglets to uninfected piglets, and the controls were transplanted with jejunal microbiota from uninfected piglets. The gene expression of Toll-like receptor (*Tlr)* 5, *Tlr*4 and *Lyz-*2 were analyzed after transplantation because previous study has shown ETEC infection significantly lower the expression of *Tlr*5, *Tlr*4 and *Lyz-*2 in the jejunum [34]. Results found that diarrheal microbiota mediated the mRNA expression of *Tlr*5 (*P* < 0.05) (*n* = 4) (Fig. 3). However, no significant difference was found about the expression of *Tlr*4 and *Lyz-*2 (*P* > 0.05) (*n* = 4) (Fig. 3). These date suggest that the dysbiosis of jejunal microbiota partly associates with the immune responses in ETEC infection.

## Discussion

Diarrhea and malnutrition are both associated with dysbiosis of the intestinal microbiota [19, 35]. ETEC is an important cause of diarrhea in humans and weaned piglets; however, the role of gut microbiota in ETEC-induced diarrhea is unknown. In current study, with different analysis methods, we found that diarrheal piglets have a dysbiosis of the intestinal microbiota, especially a higher percentage of *Lactococcus* in the jejunum, and lower *Bacteroidetes*: *Firmicutes* ratio in the jejunum and feces. Other interesting findings are that diarrheal piglets have higher percentage of *Escherichia-Shigella* and lower of *Prevotella* in the feces, and lower microbiota diversity in the jejunum and feces. There is an obvious difference about the intestinal microbiota between diarrheal piglets and resistant piglets, while little change about the intestinal microbiota is observed between resistant piglets and controls, suggesting the gut microbiota of some individuals or piglets may play a resistant role to diarrhea after exposed to inducers [16, 32, 34, 36]. A previous study found that the gut microbiota in the patients who developed diarrhea are more related to each other than to those did not develop diarrhea [36, 37]. In this study, quite part of piglets didn’t suffer from the diarrhea by the ETEC infection. Thus it seems that a specific, preexisting microbial balance might predispose or protect against diarrhea. However, piglets’ genetic resistance to ETEC has not been tested in the current study, and the jejunal microbiota from diarrheal piglets contains ETEC, it is unclear whether the ETEC in the jejunal transplant material had sufficient levels of the ETEC to cause diarrhea, which leads to cannot fully rule out the direct influence of jejunal ETEC in piglet diarrhea. Thus, the influence of intestinal microbiota on piglet diarrhea needs to further transplantation with synthetic intestinal microbiota without ETEC strain [38]. Also, further investigations are needed to explore the alterations of intestinal microbiota during ETEC induced diarrhea in piglets because this study analyzed the pre-pooled samples. Thus, to overcome the shortages of pooling samples, the intestinal microbiota was analyzed with 16S rRNA sequencing and metagenomics sequencing using different samples. The similarities from both 16S rRNA sequencing and metagenomics sequencing are considered to be the really changes in ETEC induced diarrhea. For example, although there were differences at the phylum level and genus level with each analysis, a consistent finding was that diarrheal piglets have a higher percentage of *Lactococcus lactis,* compared to the controls. Similarly, in our further experiment using bacterial counting, we found that ETEC infection increases the bacterial load of *Lactococcus lactis* in the jejunum (manuscript submitted). The discrepancy in results between 16S rRNA sequencing and metagenomics sequencing may come from various determinants, such as species, geography, and host physiology [19, 39, 40]. Indeed, the methods for analysis of intestinal microbiota also highly affect the results [41]. For example, compared to the complete 16S rRNA sequencing, sequencing of individual segments and combinations of segments greatly underestimates the taxonomic diversity [41].

ETEC-induced diarrhea is associated with a decrease in the *Bacteroidetes*:*Firmicutes* ratio. Also, a lower ratio of *Bacteroidetes*:*Firmicutes* is found in other types of diarrheal models [18, 19, 21, 33]. Thus, diarrhea, regardless of the cause, may establish an environment more suitable for survival and growth of *Firmicutes* than for *Bacteroidetes* [18, 19, 21, 33]. Previous study [18] has pointed out that the change in the *Bacteroidetes*:*Firmicutes* ratio after diarrhea is not from a change in the abundance of any particular class, but the result of a phylum-level effect. However, higher percentage of *Lactococcus* (belongs to *Firmicutes*) in diarrheal piglet jejunum, and lower percentage of *Prevotella* (belongs to Bacteroidetes) in diarrheal piglet feces may be the reason for lower *Bacteroidetes*:*Firmicutes* ratio in ETEC induced diarrhea. The exact roles of *Lactococcus* and *Prevotella* in the pathogenesis of ETEC induced diarrhea are unknown. *Lactobacillus* seems be beneficial for the recovery from ETEC induced diarrhea because *Lactobacillus reuteri* (11%)*, Lactobacillus amylovorus* (6.3%), *Lactobacillus acidophilus* (2.6%), *Lactobacillus johnsonil* (1.6%), and *Lactobacillus crispatus* (1.3%) are within the top 10 percentages of bacterium in recovered piglet jejunum. Especially, recovered piglets have higher percentage of *Lactobacillus amylovorus*, *Lactobacillus acidophilus*, and *Lactobacillus crispatus* than the diarrheal piglets.

It is unknown why a lower *Bacteroidetes*:*Firmicutes* ratio is involved in ETEC-induced diarrhea. One of the possible explanations is the intestinal level of oxygen, which can be diffused from the host tissues into the intestinal lumen [42]. After secretory stimulation (e.g., ETEC infection, Vibrio cholera infection), there is abnormally increase in the intestinal level of oxygen, which inhibits the growth of anaerobic organisms, as well as leads to the accumulation of facultative anaerobes (e.g., *Bacilli*, member of *Firmicutes*) to respire oxygen to maintain enteric anoxia [42–44]. A decrease in the relative proportion of *Bacteroidetes* is associated with various diseases, such as obesity [45]. Usually, the difference in function and metabolism between *Bacteroidetes* and *Firmicutes* is regarded as the contributor. The changed function (e.g., cell motility and genetic information processing) and metabolism (e.g., xenobiotics biodegradation and metabolism, amino acids and lipid metabolism) of the gut microbiota may be associated with the pathogenesis of diarrhea. A previous study suggested *Firmicutes* is linked to obesity because *Firmicutes* can ferment plant polysaccharide to produce short-chain fatty acids (SCFA), providing additional energy for the host [45]. Enhanced production of butyrate (SCFA) could promote the expression of globotriaosylceramide, which is a receptor for the Shiga-like toxin (Stx2), leading to increased bacterial colonization and disease severity in *Escherichia coli* O157:H7 infection [46]. Thus, the lower *Bacteroidetes*:*Firmicutes* ratio possibly could promote the attachment and colonization of pathogens (e.g., ETEC) to the intestine. A previous study found that a fat-rich diet modifies the composition of the conventional intestinal microbiota by increasing the *Firmicutes* while reducing the *Bacteroidetes* loads, creating an imbalance that exposes the intestinal epithelial cells to adherence for *Campylobacter jejuni* [47]. Thus, decreased *Bacteroidetes*: *Firmicutes* ratio may also lead to the decrease in colonization resistance against pathogens (e.g., ETEC), which promotes the colonization of pathogens in the intestine.

The gut microbiota is likely to play a pivotal role in the establishment of host-pathogen crosstalk, ultimately shaping the intestinal immune responses after infection [48–50]. Previous data have indicated that ETEC-induced diarrhea inhibits intestinal immune responses in the jejunum [34, 51]. Numerous investigations have shown that intestinal pathogens have evolved mechanisms to subvert intestinal immunity by secreting toxins to intestine after colonization [52–54]. To colonize to gut mucosal surfaces, pathogens need to inhibit intestinal immunity [53, 55]. In piglets, ETEC induced diarrhea inhibits the activation of the NF-κB pathway and MAPK pathway [34]. ETEC induced diarrhea decreases the expression of innate immune factors, including *Tlrs* [34, 56]. The inhibition of jejunal immune response in ETEC induced diarrheal piglets might be from the changed jejunal microbiota because they show decreased cell motility and flagellar assembly, which may mean decreased stimulation to the jejunum from jejunum microbiota. Flagellar filament assembly is important for flagellin expressing bacteria, such as α and ε *Proteobacteria,* to efficiently infect mammalian hosts [57, 58]. TLR5 recognizes bacterial flagellin and activates host inflammatory responses to bacteria [57, 58], thus it is not surprising to found lower expression of *Tlr*5 in diarrheal piglets compared to the controls [34]. Furthermore, jejunal microbiota transplantation from diarrheal piglets to controls, which changes the gut microbiota to diarrheal situation, also induces lower expression of *Tlr*5 compared to the controls. This supports the hypotheses that the dysbiosis of gut microbiota mediates the immune responses in ETEC induced diarrhea. Indeed, a previous study also suggested that gut microbiota is necessary for the establishment of host-pathogen crosstalk, ultimately shaping the intestinal immune responses after infection [48, 50]. However, the lack of significant change in the expression of *Tlr*4 and *Lyz-*2 after jejunal microbiota transplantation indicates that the immune responses in ETEC induced diarrhea is not fully dependent on the dysbiosis of gut microbiota, but maybe also ETEC. However, the exact function of intestinal microbiota in the immune responses in piglet-ETEC interaction needs further investigations.

## Conclusions

In conclusion, ETEC induced diarrhea is associated with the alteration of intestinal microbiota, including lower *Bacteroidetes*: *Firmicutes* ratio and microbiota diversity in the jejunum and feces, and lower *Prevotella* in the feces, but higher percentage of *Lactococcus* in the jejunum, and *Escherichia-Shigella* in the feces. Such alteration of intestinal microbiota mediates some aspects of pathogenesis in ETEC induced diarrhea. Our data also suggest there is a specific, preexisting microbial balance that predisposes or protects against ETEC induced diarrhea. It may be fruitful to attempt to treat ETEC induced diarrhea by modulating the gut microbiota.

## Additional file

Additional file 1: **S1.** A total of 6 KEGG entries were identified at KEGG level one. **S2.** A total of 37 KEGG entries were identified at KEGG level two. **S3.** A total of 236 KEGG Orthology (KO) pathways were identified at KEGG level three. (XLSX 70 kb)

## Abbreviations

cAMP: Cyclic adenosine monophosphate
cGMP: Cyclic guanosine monophosphate
ETEC: Enterotoxigenic *Escherichia coli*
KEGG: Kyoto Encyclopedia of Genes and Genomes
LT: Heat-labile toxins
ST: Heat-stable toxins
TLR: Toll-like receptor
